# Willingness to Care—Financial Incentives and Caregiving Decisions

**DOI:** 10.1002/hec.4918

**Published:** 2024-11-24

**Authors:** Mara Rebaudo, Lena Calahorrano, Kathrin Hausmann

**Affiliations:** ^1^ Fraunhofer Institute for Applied Information Technology FIT Sankt Augustin Germany; ^2^ University of Freiburg Freiburg im Breisgau Germany

**Keywords:** employment versus caregiving, long term care, social norms, SOEP, supply of informal care

## Abstract

As population aging will likely lead to an increasing number of people in need of care, the demand for informal care is expected to rise. In this context, it is often discussed whether financial incentives can motivate more individuals to assume caregiving responsibilities. We analyze the potential effect of financial incentives on the provision of informal care by estimating a structural model with endogenous labor supply and caregiving decisions. This allows us to investigate how both individual wages and financial compensations for caregiving affect the caregiving decision, while accounting for heterogeneous preferences. We find that wage increases are associated with a decreased willingness to care. Financially compensating potential carers for the opportunity costs from caregiving significantly increases the probability of providing care. However, across different subgroups, a large share of about 50% of potential carers remains unwilling to provide care despite the financial incentive. For these individuals, factors such as preferences and social norms outweigh financial considerations in their caregiving decision.

## 1 Introduction

Informal care (as opposed to care by trained professionals) is the most common form of long‐term care in many countries. For instance, in Germany, about 6% of the population had a diagnosed need for care as of 2021, and 84% of these were cared for at home, mostly by their relatives (Destatis 2022). In light of population aging, the demand for informal care is expected to increase further. The question whether this increased demand will be accommodated by informal carers, especially in times of changing family dynamics, such as lower marriage rates, fewer children, greater geographic mobility and declines in inter‐generational co‐residence (Heitmueller 2007) still remains open. In this context, it is often discussed how financial incentives, for instance leave benefits or cash‐for‐care schemes, can motivate more people to assume caregiving responsibilities. Therefore, in this paper, we analyze the potential effects of financial incentives on the caregiving decision. We consider the impact of both individual wages and financial compensations for informal care hours on the decision to provide informal care. Using a structural discrete choice model, we find that higher individual wages are associated with a lower probability of caring. Furthermore, we find large increases in the willingness to care in response to a financial compensation for the opportunity costs from care. However, even if each individual's informal care hours are compensated as paid work, about 50% of potential carers are still unwilling to provide care, suggesting that norms or preferences play an important role in the caregiving decision.

The factors influencing the willingness to care are complex and range from financial considerations regarding foregone earnings to individual and family preferences, as well as social norms (Norton 2016). How the willingness to care is related to individual wages of potential carers has first been studied by Carmichael, Charles, and Hulme (2010). They use panel data to investigate how transitions into caring depend on employment and find that being employed and earning a high wage both negatively affect the probability of getting involved in informal care. One interpretation of this finding is that potential carers foresee negative labor market consequences and avoid caregiving when their wages and hence opportunity costs are high. A different interpretation is that *higher earners* (individuals earning higher wages) may be those with lower preferences for caregiving independent of their opportunity costs. Preferences may also have affected past labor‐market choices. For instance, more family‐oriented individuals with a strong preference for caregiving may have invested less in their careers and consequently face lower opportunity costs. Additionally, social norms and expectations can play a role. For example, those not employed or earning lower wages may feel more pressure to care, while families might not expect the highest earning family member to take on caregiving. The role of opportunity costs versus norms is important, because it affects the potential impact of financial incentives on the willingness to care: If opportunity costs are important for caregiving decisions, financial incentives may have a strong impact on the willingness to care. If, instead, social norms and preferences are more relevant, then even strong financial incentives may not significantly increase the provision of informal care.

We add to this discussion by investigating how the introduction of financial incentives for informal care affects the caregiving decision of potential carers. We estimate a discrete choice model based on the German Socio‐Economic Panel (SOEP) that jointly explains the labor supply decision and the informal caregiving decision. Thereby, we account for all factors influencing potential carers' choice, including their preference for income, leisure and caregiving itself. The structural approach has the major advantage that it does not rely on specific assumptions about the complex and often unknown factors that influence caregiving decisions. Instead, the estimation of preference coefficients is based on the comparison of utility levels from different choice alternatives. Thus, the approach is useful in disentangling opportunity costs from non‐financial aspects of the caring decision.

Based on the estimated preference coefficients, we run counterfactual analyses to study how the informal care decision is affected by financial incentives. In a first step, we investigate how wages themselves affect the decision to provide care. In line with Carmichael, Charles, and Hulme (2010), we find that higher wages are associated with a lower probability of caring. Importantly, this result holds for wage increases at the individual level while accounting for differences in preferences. This implies that opportunity costs indeed matter for the caregiving decision. In a second step, we simulate a direct financial compensation for the opportunity costs from care. Specifically, all individuals receive their individual gross hourly wage for each care hour they provide. We choose this hypothetical reform scenario instead of a more realistic reform with lower replacement rates to disentangle opportunity costs from non‐financial aspects of the caring decision.

We find large increases in the willingness to care for both high and low earners in response to the financial incentive. However, a large share of about 50% of potential carers are still unwilling to provide care, implying that in many cases, non‐financial aspects are more relevant for the willingness to care than opportunity costs. Interestingly, the difference in care participation between higher and lower earners vanishes with the incentive, implying that non‐financial concerns are of similar importance in both groups. A different result is found by gender: The increase in the care participation of women and men is of similar magnitude, so that the care participation of women remains higher than that of men despite the financial incentive. This seems to confirm the existence of a social norm concerning higher caregiving responsibilities of women, whereas a norm concerning higher responsibilities of lower earners is not confirmed. An alternative financial compensation is paying each potential carer the same absolute amount, resulting in similar overall reactions, but differing results by wage groups. Specifically, the difference in care participation between higher and lower earners does not vanish when opportunity costs are not compensated equally.

The paper is structured as follows. Section 2 gives a brief description of the German institutional background and summarizes the literature on financial incentives to care. Section 3 presents the behavioral model which is the foundation for the empirical analysis. Sections 4 and 5 describe the empirical methodology and data used. Section 6 presents the estimation results of the discrete choice model, as well as the calculated elasticities and reform effects, before Section 7 concludes.

## 2 Institutional Background and Evidence on Financial Incentives for Informal Carers

### 2.1 Informal Care in Germany

In 1995, Germany introduced a long‐term care insurance (LTCI) system (BMG 2007). Currently, employees and their employers jointly contribute 3.4% of the individual gross wage to finance expenditures for care needs. Care needs were redefined in 2017, distinguishing five care levels. Care recipients are entitled to benefits if they have at least care level 2. Those cared for at home can choose between benefits in cash, benefits in kind, or a combination of both. Benefits are not means‐tested but depend on the care level of the care recipient. While benefits in kind are directly transferred to the provider of formal home care, benefits in cash are paid to the care recipient. As of 2019, the data year we use, benefits in cash ranged from 316 euros monthly for care level 2 to 901 euros for care level 5. Benefits in kind covered by LTCI range from 724 euros per month for care level 2 to 2095 euros per month for care level 5.

Caregivers whose duties last at least 10 h per week on at least 2 days are entitled to contributions to their public pension insurance system if they work no more than 30 h per week. Additionally, the Caregiver Leave Act and the Family Caregiver Leave Act regulate leaves from work for caregivers looking after close relatives. Caregivers in companies with at least 15 employees may take leaves from work to fulfill caregiving duties for up to 6 months. Caregivers in companies with at least 25 employees may take leaves for up to 24 months, where no more than 6 months can be full time leaves. When taking such a leave, caregivers are entitled to a public interest‐free loan to compensate for 50% of the temporary income losses.

Note that cash benefits for informal care can but need not be handed down to informal caregivers. Therefore, financial incentives for informal caregivers are currently relatively limited.

### 2.2 Financial Incentives for Informal Carers

The economic literature on informal care has primarily focused on the impact of caregiving on labor market outcomes, see, for instance, Carmichael, Charles, and Hulme (2010), Norton (2016), Anand, Dague, and Wagner (2022), and Frimmel et al. (2023). Bannenberg, Karlsson, and Schmitz (2019) provide a relatively recent summary of the literature on the supply of informal care. Early research, which finds negative effects on employment, assumes the informal care decision to be exogenous to the labor supply decision (Carmichael and Charles 2003). This implies that individuals would feel emotionally or morally compelled to provide care for a relative or close friend, regardless of the potential impact on their own employment. Thus, a sense of duty, rather than a free choice, would lead to the assumption of informal caregiving (Della Giusta and Jewell 2015). However, individuals may perceive at least some degree of choice in the caregiving decision. A potential carer may decide to care based on the comparison of (opportunity) costs (e.g., income losses or negative health consequences (Heger 2017; Coe and van Houtven 2009)) and benefits (e.g., positive feelings or the avoidance of guilt). Then, the decision to provide care can be seen as a rational choice, which means that caregiving and labor supply are endogenous. The endogeneity implies that observed negative effects of caregiving on employment or wages may be (partially) driven by selection effects, if those with lower wages are more likely to become caregivers. Subsequent studies try to account for this endogeneity when estimating the effects of care on employment, mostly by using instrumental variable or panel data approaches (Heitmueller 2007; van Houtven et al. 2013). The results of this research suggest that negative employment effects of caring are less severe when accounting for selection, but remain important, especially for co‐residential (those who live in the same household as the care recipient) and high‐intensity carers (those who care for 20 or more hours per week) (Heitmueller 2007). Anand, Dague, and Wagner (2022) and Frimmel et al. (2023) use health shocks experienced by the spouses or parents of potential carers, respectively, as exogenous variation. They find negative effects on the labor market outcomes of potential carers, and they find these effects to be partly mitigated by policy. The aforementioned study by Carmichael, Charles, and Hulme (2010) approaches this issue from a different perspective: Instead of viewing sample selection as a problem to resolve, they take the possibility that the willingness to care is endogenous to the labor supply decision as starting point for their research question.

The empirical evidence on the impact of financial incentives on informal care is limited and the majority of studies restricts their attention to current as opposed to potential caregivers (Bartel et al. 2023; Kang et al. 2019; Saad‐Lessler 2020). Two exceptions are Braga et al. (2022) and Costa‐Font, Jiménez‐Martín, and Vilaplana‐Prieto (2022). Braga et al. (2022) study California's paid family leave, which is found to have increased care provision for potential carers, especially women, whose parents live nearby, whereas there were no significant effects on those with a spouse in poor health. Differences in wages or opportunity costs are not studied, however. Costa‐Font, Jiménez‐Martín, and Vilaplana‐Prieto (2022) study the introduction of a caregiving allowance in Spain and find that the supply of informal care increased by 20% points in response to the reform. Despite not being means‐tested, the effects of the allowance were concentrated among lower income households. The authors argue that opportunity costs may explain why higher‐income households were less responsive to the reform. However, the data only allow for studying differences in household income of care recipients, and there is no information on individual wages of (potential) carers. Bana, Bedard, and Rossin‐Slater (2018) offer descriptive evidence on take up of paid leave for informal care in California. They find individuals with higher earnings to be overrepresented among claimants and individuals in the lowest earnings quartile to be considerably under‐represented, but do not discuss possible explanations and confounding factors.

The German LTCI's impact on labor supply (but not its impact on caregiving) has been studied by Geyer and Korfhage (2015, 2018), using SOEP data on individuals who cohabit with a person in need of care. Estimating a structural model of labor supply and of the choice of benefits, Geyer and Korfhage (2015) find that benefits in kind have a small positive impact on labor supply, while labor supply elasticities of cash benefits are larger and negative. In their subsequent research, Geyer and Korfhage (2018) use a difference‐in‐difference approach to show that the introduction of LTCI negatively impacted male labor supply but had no effect on females, likely because their participation was already low before the reform.

Summarizing, there is some recent evidence that financial incentives can increase the provision of informal care. However, it remains unclear which potential carers are more likely to react to financial incentives and why.

## 3 Behavioral Model

Traditional models of labor supply focus on the trade‐off between hours worked and leisure time. As suggested by, amongst others, Aaberge, Dagsvik, and Strøm (1995) and van Soest (1995), the discrete choice approach incorporates the labor supply decision in the context of a random utility model. The estimation of the model is based on the comparison of different utility levels instead of deriving marginal utilities over the entire set of possible working hours.^1^

Our approach extends the standard approach by modeling jointly the decision to work and the decision to get involved in informal caregiving. Specifically, we combine a set of discrete alternatives of labor supply (working hours) with discrete alternatives of informal caregiving. The structural approach allows us to account both for the role of opportunity costs, as well as for the preference for caregiving. Our approach compares to Geyer and Korfhage (2015) who also extend a structural labor supply model for informal carers by combining it with the decision between different types of benefits from the LTCI. However, while Geyer and Korfhage (2015) focus on the intensive margin among co‐residential current carers, we focus on potential carers' decision to care at the extensive margin.^2^

To study how the decision to provide care is affected by financial incentives, we focus on the group of *potential carers*, who we define in the following as all individuals who face a caregiving decision. This group consists of both actual carers, who are already engaged in caregiving, and non‐caring potential carers, who likely know someone in need of care and therefore face a caregiving decision (see Section 5 for more detail). An alternative approach would define potential carers as those who are observed to care in the future and then model repeated decision making to analyze the factors influencing the caregiving decision. However, this approach likely confounds the caring decision with the occurrence of a need for care. Additionally, it excludes those potential carers who are not observed to be carers in the future. This group is especially relevant for our research question as we are interested in the way that financial incentives affect those potential carers that do not care in the absence of further incentives. Hence, with our approach we aim to include all potential carers at one point in time and assume the need for caregiving to be exogenously determined.^3^

We assume a rational, utility‐maximizing potential carer with scarce time resources that need to be allocated between working on the labor market, caregiving, and leisure. The potential carer gains utility from income and leisure, but also directly from caregiving. The structural approach implies that the utility gained from caregiving covers all factors that influence the caregiving decision apart from individuals' preferences for income and leisure. These factors may include social norms and expectations, such as the norm that women are expected to assume caregiving responsibilities more than men, or that those with higher wages are less expected to provide care compared to potential carers with lower wages. In addition to social norms, the preference for care can be influenced by intrinsic motivation, avoidance of guilt, or even bequests and intergenerational exchange (Norton 2016). Personal values like altruism, relational skills developed through prior caregiving experiences, and the caregiver's own health and capabilities also play a role. It is also possible that the preference measures factors like the relationship with the care recipient or the difficulty of the care situation. Following the theoretical model by Johnson and Lo Sasso (2000), one could also interpret the preference for caregiving as an internalization of the preferences of the care recipient. Importantly, measured preference coefficients can also capture aversions to caregiving, such as anticipated mental or physical challenges.

The choice set of caring and non‐caring potential carers consists of three working hour categories (no work, part time and full time work) and three caregiving categories, see Table 1. The caregiving decision is a discrete choice between not caring, caring 1 h per day on average (7 h per week), or 2 h per day (14 h per week).^4^ We define *care participation* in the following as choosing one of the categories with positive care hours. While hours of work $\left(h_{w}\right)$ and informal care hours $\left(h_{c}\right)$ represent the choice categories, leisure is defined as the residual. We focus on the 7‐day week and assume 8 h of sleep, so that hours of leisure are given by the difference between total time available during the week (T=112), hours of work and hours of caregiving $\left(l=112-h_{w}-h_{c}\right)$.

**TABLE 1:** Choice set of potential carers.

	Working hours $\left(h_{w}\right)$	Informal care hours $\left(h_{c}\right)$	Leisure (l)
No work & no care	0	0	112
No work & care (low‐intensity)	0	7	105
No work & care (higher‐intensity)	0	14	98
Part time & no care	30	0	82
Part time & care (low‐intensity)	30	7	75
Part time & care (higher‐intensity)	30	14	68
Full time & no care	40	0	72
Full time & care (low‐intensity)	40	7	65
Full time & care (higher‐intensity)	40	14	58

*Note:* Each choice category is defined by a combination of weekly work and informal care hours. Leisure is the residual.

The trade‐off each potential carer is facing, can be described as follows: The decision about working hours affects income and leisure, where clearly increased working hours imply more income and less leisure time. The decision about getting involved in caregiving (independent of the labor supply decision) does not affect income, but does reduce leisure time. Which alternative is preferred by each individual is assumed to depend on the relative preferences for income, leisure and caregiving. For instance, for individuals with a high preference for leisure, the involvement in caregiving may only be considered when also reducing work hours. If, however, the income preference is high, reductions in working hours may not be considered, so that the caregiving decision depends on the relative preference for leisure and caregiving itself.

By defining the choice set this way, we directly allow for the possibility that individuals may prefer to reduce their working hours when getting involved in caregiving, which results in reduced labor supply and reduced income. Hence, potential selection in terms of carers being more likely to work fewer hours than non‐carers is directly accounted for by the empirical approach. The only assumption that we need to make is that the gross hourly wage (which is used to calculate income at the different choice alternatives) is not *causally* affected by caregiving.^5^ Importantly, it is no issue for our methodology if those with lower hourly wages in the first place are more likely to get involved in caring. In fact, the way that individual wages impact the caring decision is one aspect that we specifically analyze.

Stated mathematically, individual i chooses hours of work and hours of informal care in a way that choice alternative k is maximizing utility:

(1) $$\begin{array}{cc} U_{ik}= & U\left(C_{ik},l_{k},h_{c_{k}},\epsilon_{ik}\right)\\ = & \max_{j\in J}U\left(C_{ij},l_{j},h_{c_{j}},\epsilon_{ij}\right)\\ = & \max_{j\in J}U\left(t\left\{w_{i}h_{w_{j}},I_{i}\right\},T-h_{w_{j}}-h_{c_{j}},h_{c_{j}},\epsilon_{ij}\right) \end{array}$$

where each individual chooses from alternatives j∈J.^6^ $C_{ij}$ denotes net household income as a proxy for consumption. It consists of individual labor income (individual's gross hourly wage $w_{i}$ multiplied with working hours $h_{w_{j}}$) as well as non‐labor income and income of other household members $I_{i}$. The function t(⋅) represents the tax and transfer system applied to transform gross into net incomes. Leisure $l_{j}$ is calculated by subtracting working hours $h_{w_{j}}$ and hours spent on caregiving $h_{c_{j}}$ from total time endowment T. Time spent on caregiving $h_{c_{j}}$ enters the utility function as a separate term, as we assume that caregiving affects utility independent of its effect on leisure. Lastly, $\epsilon_{ij}$ captures unobservable tastes or disutility components for individual i when choosing alternative j. We account for these unobservable factors when calculating elasticities and reform effects in Section 6.

## 4 Econometric Specification

To estimate the random utility maximization we use a mixed logit model with random coefficients (McFadden and Train 2000). We assume a quadratic functional form of the utility function, which is common in structural labor supply models (e.g., Bargain et al. 2010; Blundell et al. 2000a, 2000b; Wrohlich 2011). Stated formally, the utility $U_{ij}$ that potential carer i gains from alternative j comprises the deterministic part $V_{ij}$ of utility and an error term $\epsilon_{ij}$ that is assumed to be iid extreme value (see Train (2009) for the econometric theory in this section).

(2) $$\begin{array}{cc} U_{ij}= & V_{ij}+\epsilon_{ij}\\ = & l_{j}\beta_{li}+C_{ij}\beta_{ci}+h_{c_{j}}\beta_{h_{c}}\\ & +l_{j}^{2}\beta_{l^{2}}+C_{ij}^{2}\beta_{c^{2}}+X_{i}^{\prime }S_{j}\beta_{X}+\epsilon_{ij}. \end{array}$$

As explained, individuals gain utility from leisure $\left(l_{j}\right)$, consumption $\left(C_{ij}\right)$, and informal care hours $\left(h_{c_{j}}\right)$. The model accounts for observed heterogeneity by interacting observable characteristics of the individuals such as age and sex $\left(X_{i}\right)$ with observable attributes of the alternatives, that is income, leisure time, hours of caregiving $\left(S_{j}\right)$. Additionally, as in Geyer and Korfhage (2015), $\beta_{li}$ and $\beta_{ci}$ are random coefficients which are allowed to vary between individuals to incorporate unobserved heterogeneity. Following Haan (2006), we assume that both random coefficients are normally distributed $\beta_{li}∼N\left(\beta_{l},W_{l}\right)$ and $\beta_{ci}∼N\left(\beta_{c},W_{c}\right)$ where the means $\beta_{l,c}$ and the variance‐covariance matrices $W_{l,c}$ are to be estimated. All other beta‐coefficients are mean coefficients. For simplicity, in the following we refer to the random coefficients as a single coefficient $\beta_{i}$.

The econometric estimation is based on comparing the utilities of the different choice alternatives: Each individual is assumed to maximize utility, implying that potential carer i will choose alternative k only if $U_{ik}>U_{ij}$ for all j≠k. Hence, the probability that individual i chooses alternative k can be formulated as

(3) $$\begin{array}{ll} P_{ik} & =\mathrm{Prob}\left(U_{ik}>U_{ij}\right)\forall j\neq k\\ & =\mathrm{Prob}\left(V_{ik}+\epsilon_{ik}>V_{ij}+\epsilon_{ij}\right)\forall j\neq k\\ & =\mathrm{Prob}\left(\epsilon_{ij}<\epsilon_{ik}+V_{ik}-V_{ij}\right)\forall j\neq k. \end{array}$$

As the error term is assumed to be iid extreme value, the logit probability conditional on $\beta_{i}$ is given by:

(4) $$L_{ik}\left(\beta_{i}\right)=\frac{e^{V_{ik}\left(\beta_{i}\right)}}{\sum_{j=1}^{J}e^{V_{ij}\left(\beta_{i}\right)}}.$$

Then, the mixed logit probability that individual i chooses alternative k is given by the integral of standard logit probabilities $L_{ik}\left(\beta_{i}\right)$ over all possible values of $\beta_{i}$:

(5) $$P_{ik}=\int L_{ik}\left(\beta_{i}\right)f\left(\beta_{i}\right)d\beta_{i}.$$

As the integral in Equation (5) does not have a closed‐form solution, simulation methods have to be used to estimate choice probabilities. Specifically, R draws for each random coefficient are taken from its assumed density. For each draw, the logit probability (4) is calculated and the results are averaged over the draws. Based on these simulated probabilities, the simulated log likelihood function takes the form:

(6) $$SLL=\sum_{i=1}^{N}\sum_{j=1}^{J}d_{ij}\;\ln\frac{1}{R}\sum_{r=1}^{R}L_{ij}\left(\beta_{i}^{r}\right)$$

where $d_{ij}$ equals one if individual i chose alternative j and zero otherwise. SLL is maximized to obtain the moments of the distribution $f\left(\beta_{i}\right)$.^7^ The derivation of individual‐level parameters and choice probabilities is described in Supporting Information S1.

## 5 Data

### 5.1 Dataset and Sample Definition

For the estimation of the model, we use data from the German Socio‐Economic Panel (SOEP), a microdata household survey run by the German Institute for Economic Research (2021). This survey gathers detailed information on personal economic circumstances, employment, preferences and personal background (Goebel et al. 2019). Each year, the SOEP data cover around 30,000 individuals in 20,000 households. For our analysis, we use data from 2019.

We restrict the sample to individuals who are between 18 and 65 years old, employable and flexible in their labor supply decision. Thereby we exclude individuals who are retired, self employed, on parental leave or in education. Most important for our research question is the definition of potential carers who face a caregiving decision. As explained in Section 3, this group consists of actual carers, who are currently engaged in caregiving activities and non‐caring potential carers, who likely face a caregiving decision. The first group of current carers is identified through the following interview question: “*What is a typical day like for you? How many hours do you spend on care and support for persons in need of care on a typical weekday, Saturday, and Sunday?*.”^8^ We define everyone who spends at least 1 h per week on informal caregiving duties as a carer. The latter group of non‐caring potential carers is more difficult to confine in the SOEP data. Ideally, we would include all individuals who have a close relative, friend or neighbor in need of care and who therefore face a caregiving decision. Unfortunately, this information is not directly available in the SOEP data. We do, however, know who shares a household with a person in need of care, and hence define those individuals as potential carers. Additionally, we can identify individuals whose partner is engaged in caregiving activities as they also likely know the person in need of care (who is being cared for by their partner). As these two groups likely only represent a fraction of non‐caring potential carers, we also include all individuals whose parents are still alive and above the age of 80.^9^

Defining our sample this way, we are left with 3581 observations. About a third of these individuals (1232) is currently engaged in caregiving, while the others are not. This proportion of about one third of potential carers being engaged in caregiving matches closely with results from other data sources.^10^

### 5.2 Net Household Income

Net household income for the different choice alternatives is calculated using a microsimulation model. This model contains detailed rules of the German tax and transfer system and is comparable to STSM and IZAΨMOD, see Steiner et al. (2012) and Löffler et al. (2014). Observed individual working hours and gross monthly wages are used to calculate hourly wages. For non‐working individuals, hourly wages are estimated following a Heckman correction procedure (Heckman 1979). Assuming that hourly wages are constant across work hour categories, gross income for each individual and each category can be calculated by multiplying the hourly wage with the number of work hours. All observable labor and non‐labor income is summed up to obtain each household's taxable income. Then, net income is calculated based on prevailing tax and transfer regulations. Hence, net household income is determined not only by taking into account all income sources, but also by household characteristics that affect taxes and transfers.

### 5.3 Sample Characteristics

Table 2 presents descriptive statistics by caregiving status. Compared to non‐caring potential carers, actual carers are more often female and younger. The age difference is likely driven by including individuals with parents above the age of 80 as non‐caring potential carers. The likelihood of having children under the age of 16 in the household does not differ significantly between carers and non‐carers. While the probability of being employed does not differ either, on average carers work about 1 h per week less than non‐carers. Additionally, carers' hourly wage is lower, while there is no statistically significant difference in net household income.

**TABLE 2:** Descriptive characteristics by caregiving status of potential carers.

	Actual carers	Non‐caring potential carers	Diff.	SE diff.
Female	0.59	0.46	0.128***	0.032
Age	47.80	53.55	−5.746***	0.842
Children in HH	0.20	0.18	0.027	0.021
Employed	0.86	0.88	−0.019	0.019
Working hours	30.35	31.64	−1.294*	0.748
Gross hourly wage	17.64	19.81	−2.176***	0.779
Net HH income	4017.76	4171.03	−153.274	141.371
Observations	1232	2349		

*Note:* Comparison of mean characteristics by caregiving status, weighted. Significance levels * p<0.10, ** p<0.05, *** p<0.01.

Note that the observed differences in employment outcomes can either be explained as a result of caregiving, or they can be the result of selection, as suggested by Carmichael et al. (2010). Therefore, Table 3 shows how the care participation differs by subgroups. We differentiate subgroups based only on exogenous variables and hence exclude hours of work or household income, which may be affected by caregiving.

**TABLE 3:** Care participation by subgroups of potential carers.

	Care participation (percent)
All	31.40
Gross hourly wage <= p50	35.82
Gross hourly wage > p50	26.99
Women	36.91
Men	25.89
Women & gross hourly wage <= p50	39.43
Women & gross hourly wage > p50	34.37
Men & gross hourly wage <= p50	28.16
Men & gross hourly wage > p50	23.60
Age <= 50	43.92
Age > 50	25.45

*Note:* Weighted shares of carers in percent under status quo conditions. Differences in caring participation between the considered subgroups are statistically significant at the 1% level for all groups except for the differences by gender and hourly wage combined, which are not statistically significant.

In the whole sample of potential carers (actual and non‐caring potential carers), about 30% are involved in caregiving. Among higher earners (with an above median gross hourly wage) the probability of caring is about 9% points lower than among lower earners, which is in line with the argument by Carmichael, Charles, and Hulme (2010) that higher wages (or social norms and preferences related to higher wages) reduce the willingness to care. For women, the probability of caring is about 11% points higher than for men. As gender is correlated with the hourly wage, we also stratify by the combination of the two: For women and men, those with a higher hourly wage have a lower likelihood to care, although these between‐group differences are not statistically significant. Lastly, in line with the age difference observed in Table 2, the care participation is about 18% points smaller for potential carers above the age of 50 compared to younger potential carers.

Summarizing, we observe that some subgroups of potential carers are more likely to care than others. To understand whether this is because of different preferences or norms or because of different opportunity costs, we estimate preference coefficients and run counterfactual analyses introducing financial incentives to care in the next section.

## 6 Results

### 6.1 Estimation Results

The estimation results of the mixed logit model are presented in Table 4. The estimated coefficients represent the effect of the different variables on utility. As explained above, random coefficients for net income and leisure are included. The standard deviation of both is statistically significant at the 1% level implying that the assumption of unobserved heterogeneity in these variables cannot be rejected.^11^ Most coefficients estimated are statistically significant. The significant coefficients on the interaction effects suggest that the preference for informal care and also for leisure varies by gender, age and children in the household. The preference for leisure additionally differs by migration background. The effect of net income varies between East and West Germans.^12^

**TABLE 4:** Estimation results—Random coefficients model.

Variables	Coefficients	
Net income	0.002***	(0.00)
Leisure	0.932***	(0.11)
Informal care	0.004	(0.03)
Net income × leisure	0.000**	(0.00)
Net income squared	−0.000***	(0.00)
Net income × East	0.001***	(0.00)
Net income × HH size > 2	0.000	(0.00)
Leisure squared	−0.004***	(0.00)
Leisure × female	0.074***	(0.01)
Leisure × age	−0.019***	(0.00)
Leisure × (age squared/100)	0.022***	(0.00)
Leisure × children in HH	0.063***	(0.01)
Leisure × adults in HH	0.005	(0.00)
Leisure × migration background	0.046***	(0.01)
Informal care × female	0.105***	(0.01)
Informal care × age	−0.005***	(0.00)
Informal care × children in HH	0.036***	(0.01)
SD		
Net income	0.001***	(0.00)
Leisure	0.136***	(0.02)
Log likelihood	−6229	
Akaike's information criterion (AIC)	12,496.827	
Observations	32,229	

*Note:* Values denote estimated coefficients. Standard errors in parentheses. The random coefficients are estimated based on simulation methods (500 pseudo‐random Halton draws). Significance levels * p<0.10, ** p<0.05, *** p<0.01.

The size of the coefficients cannot be easily interpreted due to the interaction terms. Therefore, we calculate first derivatives with respect to the three dimensions income, leisure and caregiving that can be interpreted as the respective marginal utilities. As in similar studies, the first derivative with respect to income is positive for more than 99% of all individuals. With respect to leisure, the first derivative is positive for about 92% of potential caregivers. On the one hand, negative marginal utilities of leisure can represent the intrinsic value of working. On the other hand, as explained in Geyer and Korfhage (2015), it can be expected that a part of the positive effect of leisure on utility is captured by the coefficients on informal care hours. Finally, the first derivative with respect to caregiving is positive for only about 10% of individuals. This reflects the disutility some individuals get from caregiving, for example because it is considered straining and mentally challenging. Importantly, it is not the case that the marginal utility of caregiving is positive for all actual caregivers and negative for all potential caregivers not involved in caring. In contrast, there are some caregivers for whom caregiving yields negative utility but is still considered, for example because of a negative marginal utility of leisure. Similarly, for some potential caregivers the marginal utility of caregiving is positive, but they still do not get involved in caregiving.

Once the mixed logit model is estimated, it is possible to run counterfactual analyses holding the estimated preference coefficients constant.^13^ For the calculation of elasticities and reform effects, we use the calibration method which adds a random utility component. Intuitively, it accounts for unobserved differences in utility that are not yet captured by the measured preference coefficients (see Supporting Information S1 for further information).

### 6.2 Wage Elasticities

In a first step, we calculate elasticities with respect to a 1% increase in gross wages. This is useful for validating the model, as the results of this exercise can be compared to those of structural models without an endogenous caregiving decision. Furthermore, it yields first insights into the role of opportunity costs in the caregiving decision. The elasticities presented here can be interpreted as the effect of a 1% increase in the gross hourly wage, holding all estimated preference coefficients and also the random utility component constant.

The results are presented in Table 5. The first two columns present the wage elasticities of labor force participation and total work hours respectively. For all considered subgroups, wage increases are associated with increases in labor force participation and hours worked. This suggests that on average, the substitution effect of higher wages outweighs the income effect. On average, a 1% increase in gross wages leads to an increase of labor force participation of 0.05% points, and a 0.1% increase of working hours. The labor supply effects are stronger in the lower wage group, and for women in general. These results are qualitatively and quantitatively in line with other studies using structural labor supply models based on SOEP data (e.g., Geyer and Korfhage 2015; Wrohlich 2011). For instance, Geyer and Korfhage (2015) find an increase of 0.06% points for labor force participation and a 0.18% increase for working hours. As in our results, women compared to men typically show larger labor supply reactions (e.g., Wrohlich (2011) who focuses only on mothers who care for children finds an increase in working hours by 0.49% and labor participation by 0.13% points).

**TABLE 5:** Estimated elasticities of a 1% wage increase.

	Labor participation (PP)	Working hours (%)	Informal care (PP)
All	0.050	0.103	−0.031
	(0.037, 0.065)	(0.085, 0.124)	(−0.049, −0.014)
Gross hourly wage <= p50	0.098	0.135	−0.037
	(0.072, 0.127)	(0.105, 0.169)	(−0.061, −0.015)
Gross hourly wage > p50	0.002	0.075	−0.026
	(0.001, 0.003)	(0.061, 0.091)	(−0.050, −0.003)
Women	0.091	0.167	−0.048
	(0.064, 0.120)	(0.132, 0.206)	(−0.073, −0.021)
Men	0.009	0.051	−0.016
	(0.002, 0.020)	(0.039, 0.067)	(−0.034, 0.004)
Women & gross hourly wage <= p50	0.164	0.245	−0.060
	(0.113, 0.219)	(0.179, 0.314)	(−0.094, −0.024)
Women & gross hourly wage > p50	0.018	0.103	−0.035
	(0.007, 0.031)	(0.080, 0.128)	(−0.068, −0.000)
Men & gross hourly wage <= p50	0.018	0.034	−0.009
	(0.004, 0.039)	(0.016, 0.057)	(−0.035, 0.012)
Men & gross hourly wage > p50	0.001	0.069	−0.022
	(0.000, 0.001)	(0.053, 0.085)	(−0.051, 0.007)
Age <= 50	0.038	0.092	−0.021
	(0.020, 0.058)	(0.068, 0.118)	(−0.053, 0.009)
Age > 50	0.055	0.109	−0.036
	(0.038, 0.075)	(0.086, 0.133)	(−0.055, −0.018)

*Note:* Mean Elasticities of a 1% increase in individual's gross wages. Numbers in parentheses show 90% confidence intervals obtained by parametric bootstrap with 500 draws. Differences between respective subgroups are statistically significant at the 1% level for all groups.

Most relevant to our research question are the results for informal caregiving in the third column. Overall, an increase in the gross hourly wage reduces participation in informal care, and this effect is statistically significant. This implies that, *given* the different preferences for income, leisure and caring that different individuals have, the average effect of a wage increase on the decision to provide informal care is negative. The finding by Carmichael, Charles, and Hulme (2010) that individuals with higher wages are less likely to provide informal care thus holds true at the individual level as well. Since the preference coefficients are kept constant (the preference for care does not change due to the wage increase), this result can be interpreted as initial evidence that opportunity costs indeed matter for caregiving decisions. A likely mechanism for this result is that a higher wage increases labor supply (the substitution effect outweighs the income effect), which in turn reduces leisure without further adjustments. Consequently, those with a stronger preference for leisure relative to caring are more likely to quit caring to mitigate the reductions in leisure resulting from increased employment.

The elasticity of care is negative and statistically significant for both the subgroup with below and above median wages, for women and for those above 50 years of age. For men, the estimated elasticities for care are insignificant, also when further differentiating men and women by the respective median wage. When comparing the respective subgroups (women vs. men, women with above median vs. below median wages etc.), the differences in the effects are statistically significant at the 1% level, though economically small.^14^

Summarizing, increases in the gross wage on average increase labor force participation and working hours and decrease informal care participation. Importantly, we find that these effects hold at the individual level when accounting for preferences, which we interpret as evidence that opportunity costs are relevant for the caregiving decision. To gain further insights into the role of opportunity costs as opposed to social norms and preferences, in the next step, we simulate a hypothetical “reform” that financially compensates informal care with each individual's hourly wage.

### 6.3 Financial Incentives to Care

In this section we analyze the impact of a direct financial incentive to care on the decision to become involved in caregiving. As discussed in the previous sections, higher earners are less likely to participate in caregiving compared to lower earners, either because of their higher opportunity costs, *or* because of different preferences or societal expectations. To shed light on these different motives, we simulate a hypothetical reform that financially compensates individuals for the opportunity costs from caring. Specifically, each individual's informal care hours are compensated with their individual gross hourly wage regardless of their labor supply.^15^ This gross compensation for care hours is treated as additional labor income in the calculation of net household income. As a result, there is no financial difference between working or caring for one additional hour.

Consequently, we expect positive effects on the caring decision from those individuals for whom opportunity costs are the main reason for not being involved in caregiving. In contrast, if the reason for differences in the participation in care across different subgroups is not opportunity costs but mainly preferences and norms, we expect no strong positive impact of the reform on the willingness to care. Of course, the two factors are potentially linked and the results depend on the relative preference for income, leisure and care: For example, if the income preference is high and the leisure preference is low, then financial incentives may be strong enough to compensate for negative marginal utilities from caring. However, for some potential carers the preference against caregiving may be too high for the caring decision to be affected by the provided financial incentive. These individuals will not be willing to become involved in caregiving despite the large financial incentive.

Table 6 presents the results for this simulation. For all considered subgroups the reform has a large positive effect on informal care participation and a smaller negative effect on labor supply. Overall, the reform increases participation in informal care by about 18% points. The estimated post‐reform care participation is 47% (see Table 7). Thus, financial incentives can affect the willingness to care. However, a large proportion of about one half of potential carers remains unwilling to provide informal care, even when informal care hours are fully compensated as paid work. This reflects the importance of non‐financial factors in the caregiving decision.

**TABLE 6:** Estimated effects of compensating informal care as paid work.

	Labor participation (PP)	Working hours (%)	Informal care (PP)
All	−2.183	−2.968	18.446
	(−2.540, −1.859)	(−3.378, −2.555)	(16.106, 20.786)
Gross hourly wage <= p50	−2.874	−3.934	12.441
	(−3.425, −2.353)	(−4.614, −3.264)	(10.844, 14.114)
Gross hourly wage > p50	−1.489	−2.123	24.475
	(−1.697, −1.282)	(−2.412, −1.813)	(21.466, 27.376)
Women	−3.602	−4.185	17.238
	(−4.215, −3.032)	(−4.932, −3.486)	(15.194, 19.336)
Men	−0.783	−1.975	19.638
	(−1.095, −0.467)	(−2.369, −1.584)	(16.858, 22.381)
Women & gross hourly wage <= p50	−4.718	−6.137	13.038
	(−5.673, −3.845)	(−7.338, −4.996)	(11.427, 14.789)
Women & gross hourly wage > p50	−2.483	−2.586	21.446
	(−2.884, −2.151)	(−3.041, −2.137)	(18.960, 23.910)
Men & gross hourly wage <= p50	−0.807	−2.150	11.958
	(−1.146, −0.490)	(−2.587, −1.710)	(10.254, 13.764)
Men & gross hourly wage > p50	−0.760	−1.806	27.350
	(−1.090, −0.430)	(−2.186, −1.425)	(23.551, 30.978)
Age <= 50	−4.358	−5.485	20.698
	(−5.078, −3.611)	(−6.378, −4.572)	(18.497, 22.863)
Age > 50	−1.128	−1.806	17.355
	(−1.490, −0.797)	(−2.203, −1.445)	(14.844, 19.861)

*Note:* Mean effects of a reform that compensates informal care hours with each individual's gross hourly wage. Numbers in parentheses show 90% confidence intervals obtained by parametric bootstrap with 500 draws. Differences in effects between respective subgroups are statistically significant at the 1% level for all groups.

**TABLE 7:** Care participation in response to compensating informal care by individual wage.

	Care participation (percent)
All	47.00
Gross hourly wage <= p50	44.65
Gross hourly wage > p50	49.35
Women	51.01
Men	43.04
Women & gross hourly wage <= p50	49.41
Women & gross hourly wage > p50	52.61
Men & gross hourly wage <= p50	36.92
Men & gross hourly wage > p50	49.18
Age <= 50	61.39
Age > 50	40.02

*Note:* Weighted shares of carers in percent in response to reform that compensates informal care as paid work. Differences in post‐reform care participation between the respective subgroups are statistically significant at the 1% level for all groups. Post‐reform participation differs from the sum of status quo participation and reform effects because calibration procedure does not yield valid estimates for all observations.

Comparing the estimated reform effects by wage groups shows that for the group with above median wages, the impact on informal care is about 12% points larger than in the lower wage group. Thus, among higher earners, more potential caregivers are induced to participate in informal caregiving when they receive financial compensation. However, considering the lower participation in caregiving among higher earners before the reform (Table 3), there is also greater potential to increase participation in this group. Note that after the reform, caregiving participation is similar in both groups, and even slightly higher in the higher wage group (49% for higher earners compared to 45% for lower earners). This suggests that the unwillingness to provide care that is unexplained by opportunity costs—and hence due to preferences or social norms—is of similar importance in both wage groups. Relating back to the study by Carmichael et al. (2010), this implies that higher earners are indeed less likely to provide care *because* of their opportunity costs: When accounting for opportunity costs, the gap in caregiving participation between higher and lower earners vanishes.

Importantly, the difference between wage groups is not merely a gender effect: Stratifying by both gender and wage shows that the difference in the estimated reform effect between lower and higher‐wage groups exists for both women and men. Hence, the above result that higher‐wage groups react more strongly to the reform is not driven by the gender difference in wages. Among women, the gap in care participation between higher and lower earners vanishes after the reform with even a slightly higher post‐reform care participation for women in the higher‐wage group (53% for female higher earners and 49% for female lower earners). For men, the stronger reaction among those with higher wages results in a significantly higher post‐reform care participation compared to men with lower wages (49% for male higher earners and 37% for male lower earners). Hence, for men it seems like the unwillingness to provide care that remains unexplained by opportunity costs is larger among lower earners.

A different result is found when stratifying by gender and age groups: In our sample of actual and non‐caring potential carers, the share of carers under status quo conditions is considerably higher among females and younger individuals. Despite small differences in the reaction to the reform between the respective groups, the post‐reform care participation remains significantly higher for females and for individuals below the age of 50. For example, after the reform, men are still 8% points less likely to be involved in caregiving than women, and this difference is statistically significant at the 1% level (see Supporting Information S1: Table A4). This suggests that preferences and norms, relative to opportunity costs, are more relevant for men and older individuals compared to women and younger potential carers. However, post‐reform care participation of females is still only around 50%, while it is approximately 61% for young potential carers. Thus, even within these two groups a significant unwillingness to care persists when informal care is financially compensated.

Summarizing, financial incentives to care do induce large increases in the informal care participation. However, even compensating informal care as paid work does not induce a willingness to care for everyone. This remaining unwillingness to care is driven by either norms or preferences. While norms and preferences cannot be analyzed separately in this context, several conclusions can be drawn from the results: Based on the literature, we assume that two important social norms related to the provision of care may be relevant: Women (and not men) are expected to be the primary caregivers, and so are lower as opposed to higher earners. Since the difference in care participation between higher and lower earners vanishes when accounting for opportunity costs, it is indeed the opportunity costs that explain the difference in the status quo care participation, and any remaining unwillingness to care is driven by preferences that do not differ systematically by group‐specific norms. A different result is found for the norm concerning gender: When opportunity costs are accounted for, women are still more likely to be involved in caregiving than men. Thus, there seem to be important differences in the non‐financial aspects concerning caregiving decisions between women and men. We assume that the norm assigning primary caregiving responsibility to women indeed plays a crucial role in the remaining difference in the caregiving participation.

For the results presented in Table 6 it is important to note that, in absolute terms, the financial compensation is higher for higher earners. Hence, the financial incentives are different from those of policies that compensate each potential carer by a replacement rate below 100% or even by the same amount. For example, Costa‐Font, Jiménez‐Martín, and Vilaplana‐Prieto (2022) find that reactions to the introduction of a fixed caregiving allowance in Spain were concentrated among lower income groups. For our research question, namely disentangling opportunity costs from non‐financial aspects of the caring decision, it is more helpful to consider a financial incentive that fully accounts for differences in wages and thus in potential opportunity costs. To illustrate the difference, we provide the simulation results of an alternative reform with a fixed benefit in Table 8. The reform is defined as revenue‐neutral compared to the reform simulated above. Thus, we calculate the mean net compensation of the above reform and add this fixed amount to the household income for each of the choice alternatives with a participation in caregiving. The opportunity costs of caring are thus mitigated by the financial compensation, but remain higher for higher earners. The average impact of introducing this kind of financial compensation is similar to that of the reform in Table 6, resulting in a post‐reform care participation of about 48% (see Supporting Information S1: Table A5). While the difference in response effects between the respective subgroups is statistically significant, it is much smaller than for the reform compensating each by their individual wage. Additionally, with this reform the groups with higher status quo participation (e.g., lower earners and women) are the ones with larger increases in their care participation. As a consequence, the gap in the status quo care participation between the respective groups remains statistically significant in response to this reform (see Supporting Information S1: Table A6).

**TABLE 8:** Estimated effects of compensating informal care by average wage.

	Labor participation (PP)	Working hours (%)	Informal care (PP)
All	−3.454	−4.356	19.916
	(−4.114, −2.846)	(−5.093, −3.612)	(17.314, 22.466)
Gross hourly wage <= p50	−5.585	−7.381	22.161
	(−6.798, −4.482)	(−8.818, −6.028)	(19.240, 25.205)
Gross hourly wage > p50	−1.315	−1.710	17.663
	(−1.522, −1.133)	(−1.952, −1.460)	(15.450, 19.729)
Women	−5.762	−6.744	22.283
	(−6.947, −4.733)	(−8.213, −5.481)	(19.568, 25.008)
Men	−1.177	−2.410	17.582
	(−1.596, −0.813)	(−2.934, −1.911)	(15.114, 20.141)
Women & gross hourly wage <= p50	−8.873	−11.599	24.427
	(−11.016, −7.057)	(−14.318, −9.260)	(21.347, 27.652)
Women & gross hourly wage > p50	−2.645	−2.764	20.135
	(−3.116, −2.256)	(−3.307, −2.297)	(17.729, 22.487)
Men & gross hourly wage <= p50	−1.907	−3.846	19.497
	(−2.614, −1.312)	(−4.719, −3.022)	(16.618, 22.507)
Men & gross hourly wage > p50	−0.443	−1.019	15.659
	(−0.640, −0.279)	(−1.261, −0.799)	(13.454, 17.866)
Age <= 50	−6.115	−7.474	23.195
	(−7.172, −5.096)	(−8.797, −6.278)	(20.738, 25.699)
Age > 50	−2.163	−2.916	18.327
	(−2.848, −1.550)	(−3.705, −2.224)	(15.610, 21.060)

*Note:* Mean effects of a reform that compensates informal care hours with the average net benefit of the compensation by individual hourly wages. Numbers in parentheses show 90% confidence intervals obtained by parametric bootstrap with 500 draws. Differences in effects between respective subgroups are statistically significant at the 1% level for all groups.

## 7 Discussion

Informal care is a central pillar of long‐term care systems in many countries. Therefore, an important political discussion concerns the question whether the expected increase in the demand for informal care due to population aging can be accommodated by informal carers and whether financial incentives for potential carers are effective in increasing the provision of informal care.

We address this question using a structural discrete choice model, where both labor supply and caregiving are endogenous. We find that increases in the gross hourly wage decrease the willingness to care across different subgroups, suggesting that opportunity costs are important for the caregiving decision. This implies that the supply of informal care may even decrease in the future, especially when female labor force participation and wages increase further. The introduction of a financial compensation for caring yields large increases in the caring participation across wage groups, age groups, and both genders. However, even compensating informal care hours as paid work does not induce all potential carers to provide care. In fact, about half of all potential carers remain unwilling to care despite the incentive. This suggests that preferences against caregiving, as well as societal norms and expectations, are more relevant than financial considerations for a sizable part of potential carers. Interestingly, the gap in the caring participation between higher and lower earners vanishes with the wage‐dependent financial compensation, suggesting that the unwillingness to care that is driven by non‐financial aspects is of similar importance for higher and lower earners. Results by gender are different, as the care participation remains higher for women compared to men even when opportunity costs are accounted for. Thus, gender norms concerning the provision of care seem to be more relevant than norms concerning higher versus lower earners. While our structural approach is well‐suited to disentangle opportunity costs from non‐financial aspects of the caring decision, it does not provide direct insights into the underlying complex factors influencing the willingness to care. Future research could delve deeper into these factors to investigate which non‐financial aspects are relevant for caregiving decisions.

Our study has a few limitations. Firstly, assumptions need to be made for the definition of potential carers, as we do not directly observe whether individuals have relatives or other acquaintances in need of care. Secondly, we can only analyze short‐term and thus partial‐equilibrium effects. If financial incentives induce a higher care participation for demographic groups who are currently less involved in caregiving, such as men and higher earners, preferences for informal care and social norms may change over time. For instance, norms implying caregiving responsibilities for women versus men may lose in importance. This in turn may make financial incentives more effective but also less necessary. If the supply of informal care increases, continuing to provide financial incentives may prove costly and thus less attractive, and in general equilibrium there may be some “optimal” amount of financial incentives.

Finally, it is important to note that we do not suggest that more informal care is always a desirable outcome. Previous research has demonstrated that informal care can have both positive and negative impacts on both caregivers and care recipients (Bauer and Sousa‐Poza 2015). However, particularly for countries heavily dependent on informal care, the expected increase in demand will bring significant challenges. Moreover, involving more individuals in caregiving responsibilities—potentially with fewer individual hours—may help mitigate the adverse effects on individual caregivers. Our findings imply that financial incentives alone may not be sufficient to motivate potential carers in the future.

## Conflicts of Interest

The authors declare no conflicts of interest.

## Supporting information

Supporting Information S1

## Data Availability

The data that support the findings of this study are available from DIW Berlin. Restrictions apply to the availability of these data, which were used under license for this study. Data are available from https://doi.org/10.5684/soep.core.v36eu with the permission of DIW Berlin.

## Endnotes

^1^
With respect to work hours, the discrete choice approach takes into account that observed working hours are concentrated at particular points of the distribution, such as part‐time and full‐time work. Furthermore, it allows to model nonlinearities in the budget constraint resulting from regulations of the tax‐benefit system (Aaberge and Colombino 2018).

^2^
Other examples that extend labor supply models by including several dimensions of choice are Kornstad and Thoresen (2007) and Wrohlich (2011) who model the simultaneous choice of labor supply and child‐care and Haan and Wrohlich (2011) who analyze employment and fertility decisions.

^3^
Clearly, whether a need for informal care arises will be pivotal in determining an individual's decision to get involved in caring (Carmichael, Charles, and Hulme 2010). However, here we abstract from the care recipient's need and focus on an individual's willingness to care. As we are not restricting our attention to high‐intensity caregiving, we assume that care is provided for the care recipient (by professional caring services or by other relatives or friends) and that individuals decide whether to provide additional care. This could either imply increased overall care or some reduction in care from the other care providers.

^4^
The choice categories of 7 or 14 h of care were selected because they represent the two most frequently reported hours among actual carers in our sample. The estimated effects are similar for alternative representations of the choice set, see Supporting Information S1: Table A2 for the main reform. The most frequently chosen hour levels may change in reality if a reform, as analyzed in the following sections, were to be implemented. This reaction, however, cannot be modeled, as the structural approach limits the number of possible categories and requires the same hour levels for both the status quo and the reform.

^5^
Similarly, it is common practice in labor supply models to assume that the gross hourly wage is constant across working hour categories. While the overall evidence for causal effects of caring on hourly wages is mixed, especially due to econometric challenges, the assumption seems to be justified at least for lower‐intensity caregiving (Bannenberg, Karlsson, and Schmitz 2019). For Germany, Schmitz and Westphal (2017) find no significant effects of caring on gross hourly wages at least in the short‐run.

^6^
Following Geyer and Korfhage (2015) and Wrohlich (2011), we do not model joint decision making for couples and instead treat the partners' labor‐care‐decision as given. In our case this simplifying assumption is made for two reasons: First, the number of observations of potential working‐age carers in our sample is too small to increase the number of choice alternatives for joint decision making and for separately studying couples and singles. Second, the caregiving decision should not (only) depend on the partner's behavior, but more importantly on the decisions of other individuals caring for the same care recipient (e.g., siblings or other relatives living in other households). With the SOEP data it is, however, not possible to identify other extra‐residential carers for the same care recipient.

^7^
For estimation, we use the Stata ado mixlogit based on Halton draws, described by Hole (2007). Following Geyer and Korfhage (2015), we do not allow the random parameters to be correlated.

^8^
Child care is listed as a separate activity in this time‐use question, implying that confusion with different caring activities is unlikely. Furthermore, the given definition of informal care likely excludes housework activities (washing, cooking, cleaning) as these are also separately listed as activities.

^9^
Estimated effects of the main reform are similar when instead only including those with parents above the age of 80 as non‐caring potential carers, see Supporting Information S1: Table A3.

^10^
In the SOEP‐IS data from 2016 respondents were asked if they know a person in need of care, see Richter and Schupp (2015) and Ehrlich and Kelle (2019).

^11^
For alternative model specifications with random coefficients for informal care, convergence could not be achieved. Note, however, that unobserved heterogeneity in the preference for caregiving can partly be captured by the random coefficients for leisure and, additionally, unobserved heterogeneity is introduced into the model by calibrating a vector of error terms.

^12^
Despite not using any alternative‐specific constants, the model fits the data well with no systematic over‐ or underprediction of any of the alternatives, see Supporting Information S1: Table A1.

^13^
In reality, preferences may change over time, which could also be induced by reforms affecting the willingness to care. As is typical with this methodology, here, we focus on short‐run effects that abstract from the possibility of changing preferences.

^14^
Results of the t‐tests are available upon request.

^15^
For the purpose of disentangling opportunity costs from norms and preferences, this is more helpful than simulating a more realistic policy with a replacement rate below 100%.
