# Supplementary material for: Willingness to Care—Financial Incentives and Caregiving Decisions
Source: Health Econ. 2024 Nov 24;34(3):442–55. doi: 10.1002/hec.4918 (PMC11786941; doi:10.1002/hec.4918)
Supplement: Supplementary file 1 — Supporting Information S1 [file HEC-34-442-s001.pdf]

Appendix

Model Fit

In Table A1, we compare observed probabilities for each choice category with the in-sample prediction of the mixed logit model. Even though we do not use any alternative-specific constants, the model fits the data well with no systematic over- or underprediction of any of the alternatives.

Table A1: Within-sample Fit. Observed vs. Predicted Probabilities.

	Observed	Predicted
No work & No Care	9.55	8.28
No work & Care (low-intensity)	4.08	6.47
No work & Care (higher-intensity)	6.45	5.92
Part time & No Care	24.63	23.04
Part time & Care (low-intensity)	6.39	7.96
Part time & Care (higher-intensity)	5.33	3.41
Full time & No Care	31.42	31.38
Full time & Care (low-intensity)	8.13	9.71
Full time & Care (higher-intensity)	4.02	3.84

Notes: The table compares the observed probabilities with the choice probabilities predicted by the mixed logit model. The values are given in percent.

Individual-level Parameters

The estimation of individual-specific parameters follows the approach suggested by Revelt and Train (2000). The general idea of this approach is based on the assumption that the distribution of preferences among individuals who make a particular choice differs from the distribution of preferences over the entire population. Conditional on attributes and choice decision a distribution of β_i can be derived, that can be used to estimate individual parameters. Based on this, individual level specific choice probabilities can be estimated that are used to calculate individual derivatives and elasticities.

Deriving Elasticities and Reform effects

For the calculation of elasticities or reform effects in structural labor supply models, one can distinguish between two common methods (Creedy and Duncan, 2002): The "probability" method, among others applied by Geyer and Korfhage (2015), is based on the comparison of the predicted probabilities for each alternative before and after a wage change or policy reform. The relative changes, for example in the predicted working hours, are then interpreted as elasticities. The major disadvantage of this method is that it does not make use of the information on the actually chosen alternative under status quo conditions. Thereby, it ignores the utility-component that remains unexplained by the model. Therefore, in this paper we use the alternative method, the so called "calibration"

technique (Duncan and Weeks, 1998; Creedy and Kalb, 2005). As explained in detail by Creedy and Kalb (2005), the idea is to draw error terms from the extreme value distribution that result in the observed alternative chosen being the optimal choice for each individual. This calibrated vector of error terms is then added to the deterministic part of utility for each alternative to calculate the new optimal alternative after a wage change (or policy reform). To gain robust elasticity estimates, this process is repeated for a sufficient number of times – in this case 100. By averaging the predicted choices over the 100 repetitions, individual probabilities for being in each of the alternatives can be calculated. These can be compared to the actual choice of alternatives under status-quo conditions to determine the effect of the policy reform.

We interpret this calibrated vector of error terms as unobserved utility component of each alternative choice. Hence, it captures additional unobserved heterogeneity in preferences that is not yet captured by the estimation of the mixed logit model. For our research question this unobserved utility component is especially relevant, as it may cover factors like social norms or societal expectations with regard to the caring decision.[1]

Confidence intervals for the elasticities are obtained by a parametric bootstrap as suggested by Krinsky and Robb (1986). The basic idea is that once the mean and variance of the parameter vector $\hat{\beta}$ are consistently estimated, then the mean and variance of a function of $\hat{\beta}$ can be estimated (Greene, 2012). Hence, we use the mean and covariance of the estimated coefficients of the mixed logit model to draw 500 new coefficients. For each of the 500 draws, the calibration procedure is applied to calculate elasticities. Following the standard approach in the literature we use these elasticities to construct 90% confidence intervals.

[1]These factors are already covered by the estimated preference coefficients to the extent that they can be represented through the defined interaction terms and random coefficients for income and leisure. However, the resulting coefficients do not result in the observed alternative chosen being the optimal choice for each individual. This implies that there are additional factors influencing each individual's choice that we can account for by calibrating the vector of error terms as described.

Sensitivity Analyses

Table A2: Estimated effects of compensating informal care as paid work. Alternative definitions of the choice set.

	Labor Participation (PP)	Working Hours (%)	Informal Care (PP)
**Care hours 0 - 7**			
All	-2.050	-2.556	15.176
	(-2.525, -1.667)	(-3.044, -2.152)	(12.951, 17.289)
Gross Hourly Wage <= p50	-3.054	-3.933	11.047
	(-3.815, -2.412)	(-4.837, -3.154)	(9.381, 12.720)
Gross Hourly Wage > p50	-1.042	-1.371	19.326
	(-1.260, -0.871)	(-1.608, -1.189)	(16.645, 21.979)
**Care hours 0 - 10 - 20**			
All	-2.745	-3.477	18.639
	(-3.168, -2.335)	(-4.008, -2.915)	(16.295, 20.911)
Gross Hourly Wage <= p50	-3.087	-4.191	12.081
	(-3.710, -2.450)	(-5.010, -3.348)	(10.468, 13.622)
Gross Hourly Wage > p50	-2.401	-2.891	25.215
	(-2.703, -2.131)	(-3.298, -2.461)	(22.149, 28.344)
**Care hours 0 - 5 - 10 - 20**			
All	-2.994	-3.613	18.200
	(-3.493, -2.516)	(-4.189, -3.049)	(15.690, 20.461)
Gross Hourly Wage <= p50	-3.861	-4.814	12.933
	(-4.625, -3.141)	(-5.775, -3.953)	(11.047, 14.724)
Gross Hourly Wage > p50	-2.125	-2.545	23.475
	(-2.412, -1.836)	(-2.884, -2.184)	(20.390, 26.413)

Notes: Mean effects of a reform that compensates informal care hours with each individual's gross hourly wage. Alternative specifications of the choice set for informal care hours. Numbers in parentheses show 90% confidence intervals obtained by parametric bootstrap with 500 draws.

Table A3: Estimated effects of compensating informal care as paid work. Alternative definition of potential carers.

	Labor Participation (PP)	Working Hours (%)	Informal Care (PP)
All	-1.661	-2.559	18.221
	(-2.022, -1.352)	(-2.973, -2.157)	(15.698, 20.767)
Gross Hourly Wage <= p50	-2.496	-3.554	11.800
	(-3.095, -1.994)	(-4.247, -2.910)	(10.209, 13.501)
Gross Hourly Wage > p50	-0.825	-1.647	24.660
	(-0.984, -0.665)	(-1.914, -1.391)	(21.373, 28.034)
Women	-2.994	-3.629	16.151
	(-3.598, -2.450)	(-4.306, -2.969)	(14.021, 18.339)
Men	-0.263	-1.607	20.395
	(-0.430, -0.132)	(-1.892, -1.349)	(17.446, 23.473)
Women & Gross Hourly Wage <= p50	-4.127	-5.182	11.384
	(-5.071, -3.313)	(-6.322, -4.165)	(9.820, 13.056)
Women & Gross Hourly Wage > p50	-1.858	-2.274	20.929
	(-2.267, -1.479)	(-2.752, -1.821)	(18.234, 23.686)
Men & Gross Hourly Wage <= p50	-0.470	-1.964	12.689
	(-0.781, -0.231)	(-2.374, -1.626)	(10.815, 14.808)
Men & Gross Hourly Wage > p50	-0.054	-1.256	28.122
	(-0.097, -0.024)	(-1.520, -1.010)	(24.212, 32.310)
Age <= 50	-2.950	-3.965	18.743
	(-3.561, -2.415)	(-4.632, -3.342)	(16.718, 20.714)
Age > 50	-1.097	-1.975	17.993
	(-1.465, -0.770)	(-2.370, -1.590)	(15.350, 20.778)

Notes: Mean effects of a reform that compensates informal care hours with each individual's gross hourly wage. Potential carers defined as actual carers and individuals with parents above the age of 80. Numbers in parentheses show 90% confidence intervals obtained by parametric bootstrap with 500 draws.

Post-Reform Care Participation and Statistical Tests of Differences

Table A4: Difference in Care Participation in Response to Compensating Informal Care by Individual Wage.

	Difference in Care Participation
Gross Hourly Wage > p50	4.699***
vs. Gross Hourly Wage <= p50	(0.092)
Men	-7.971***
vs. Women	(0.091)
Women & Gross Hourly Wage > p50	3.201***
vs. Women & Gross Hourly Wage <= p50	(0.081)
Men & Gross Hourly Wage > p50	12.254***
vs. Men & Gross Hourly Wage <= p50	(0.110)
Age > 50	-21.365***
vs. Age <= 50	(0.089)

Notes: T-tests for comparison of post-reform care participation between subgroups. Reform that compensates informal care hours with each individual's gross hourly wage. SE diff. in parentheses. Significance levels * $p < 0.10$, ** $p < 0.05$, *** $p < 0.01$.

Table A5: Care Participation in Response to Compensating Informal Care by Average Wage

	Care Participation (percent)
All	48.47
Gross Hourly Wage <= p50	54.37
Gross Hourly Wage > p50	42.54
Women	56.05
Men	40.98
Women & Gross Hourly Wage <= p50	60.80
Women & Gross Hourly Wage > p50	51.30
Men & Gross Hourly Wage <= p50	44.46
Men & Gross Hourly Wage > p50	37.49
Age <= 50	63.88
Age > 50	40.99

Notes: Weighted shares of carers in percent in response to reform that compensates informal care by average wage. Differences in post-reform care participation between the respective subgroups are statistically significant at the 1% level for all groups. Post-reform participation differs from the sum of status quo participation and reform effects because calibration procedure does not yield valid estimates for all observations.

Table A6: Difference in Care Participation in Response to Compensating Informal Care by Average Wage.

	Difference in Care Participation
Gross Hourly Wage > p50	-11.832***
vs. Gross Hourly Wage <= p50	(0.098)
Men	-15.072***
vs. Women	(0.100)
Women & Gross Hourly Wage > p50	-9.499***
vs. Women & Gross Hourly Wage <= p50	(0.107)
Men & Gross Hourly Wage > p50	-6.977***
vs. Men & Gross Hourly Wage <= p50	(0.097)
Age > 50	-22.891***
vs. Age <= 50	(0.098)

Notes: T-tests for comparison of post-reform care participation between subgroups. Reform that compensates informal care hours with average wage. SE diff. in parentheses. Significance levels * $p < 0.10$, ** $p < 0.05$, *** $p < 0.01$.

References

Creedy, J. and A. Duncan (2002). Behavioural Microsimulation with Labour Supply Responses. *Journal of Economic Surveys 16*(1).

Creedy, J. and G. Kalb (2005). Discrete Hours Labour Supply Modelling: Specification, Estimation and Simulation. *Journal of Economic Surveys 19*(5).

Duncan, A. and M. Weeks (1998). Simulating Transitions using Discrete Choice Models. *Publications of the American Statistical Association 106*, 151–156.

Geyer, J. and T. Korfhage (2015). Long-term Care Insurance and Carers' Labor Supply - A Structural Model. *Health economics 24*(9), 1178–1191.

Greene, W. H. (2012). *Econometric Analysis* (7th ed. ed.). Boston: Prentice Hall.

Krinsky, I. and L. A. Robb (1986). On Approximating the Statistical Properties of Elasticities. *The Review of Economics and Statistics 68*(4), 715–719.

Revelt, D. and K. Train (2000). Customer-Specific Taste Parameters and Mixed Logit: Households' Choice of Electricity Supplier. *Working Paper, Department of Economics, UCB* (No. E00-274).
